# Day‐to‐day blood pressure variability and severity of COVID‐19: Is sympathetic overdrive a potential link?

**DOI:** 10.1111/jch.14337

**Published:** 2021-07-30

**Authors:** Michiaki Nagai, Takeshi Fujiwara, Kazuomi Kario

**Affiliations:** ^1^ Department of Cardiology Hiroshima City Asa Hospital Hiroshima Japan; ^2^ Division of Cardiovascular Medicine Department of Medicine Jichi Medical University School of Medicine Tochigi Japan

The paper by Li and coworkers^1^ in this issue of the *Journal* provides several new insights into the significant relationships of three parameters in day‐to‐day SBP variability–that is, standard derivation [SD], coefficient of variation [CV], and variability independent of mean [VIM]–with worse clinical outcomes in 79 hospitalized patients with COVID‐19.^1^

Coronavirus disease 2019 (COVID‐19), which is caused by severe acute respiratory syndrome coronavirus 2 (SARS‐CoV‐2) and currently a worldwide pandemic, includes in its pathophysiology an excessive inflammatory phase called a “cytokine storm” that is closely linked to its high mortality.^2^ This storm leads to activation of immune cells, release of inflammatory cytokines, and recruitment of further cells of the immune system.^3^ When this immune response is exaggerated, excessive inflammation leads to end tissue damage and organ failure. In general, the cytokine storm is thought to be caused by an imbalance of the autonomic nervous system (ANS),^4^ which is linked to overexcitation of the illness‐adaptive sympathetic nervous system (SNS) to maintain homeostasis.^4^, ^5^ Indeed, an imbalance of the ANS has been suggested to determine the severity of courses in COVID‐19.^6^

While hypertension is an important predictor of cardiovascular disease (CVD),^7^ blood pressure (BP) fluctuation is partially caused by changes in internal physical status. In the UK‐TIA Aspirin Trial and the ASCOT‐BPLA, visit‐to‐visit systolic BP (SBP) variability was shown to be a strong predictor of stroke independent of average SBP.^8^ Day‐to‐day home SBP variability was also associated with the poor prognosis of the Japanese at high risk of CVD.^9^

In a recent analysis of COVID‐19, a significant association between higher BP levels at admission and higher mortality was demonstrated,^10^ and poor BP control was also found to be independently associated with a higher risk of an adverse outcome in COVID‐19.^11^ Thus, Li and coworkers^1^ added increased day‐to‐day BP variability as a non‐conventional risk factor for poor prognosis in hospitalized patients with COVID‐19.

Several mechanisms have been considered to play roles in the pathophysiology of COVID‐19 including systemic inflammation, disrupted cardiopulmonary coupling, and cardiac insufficiency to compromise BP regulation, and any of these mechanisms could potentially lead to the increased BP variability that precedes the deterioration of general condition in some patients with COVID‐19.^1^ Although the causes of abnormal BP variability are still under debate, an autonomic factor specifically, SNS overactivity is likely to be involved.^12^

As mentioned above, SARS‐CoV‐2 infection induces an excessive immune reaction leading to a fatal cytokine storm and multi‐organ failure. This storm involves the release of considerable amounts of proinflammatory cytokine including IL‐6 and TNF‐α.^13^ These mediators can subsequently cross the blood brain barrier, ultimately increasing SNS activation via dysregulation of the central autonomic network comprising the insular cortex, anterior/mid‐cingulate cortices, amygdala, hypothalamus, periaqueductal gray matter, parabrachial complex, nucleus of the tractus solitarius, and rostral ventrolateral medulla.^4^, ^14^, ^15^ Increased activation of the resting and reactive SNS could have detrimental effects on several physiological systems, including alterations in cardiac contraction,^16^ impairments in vascular function,^17^ and reductions in exercising blood flow capacity.^18^

The increased SNS discharge is well known to be associated with hypoxic condition that increases peripheral chemosensitivity.^19^ Chronic obstructive pulmonary disease has been shown to increase SNS activation mainly through chronic hypoxia, which acts by increasing the peripheral chemosensory response.^20^ In a sub‐study of the Japan Morning Surge‐Home Blood Pressure Study, decreased respiratory function assessed by percent vital capacity was associated with exaggerated ambulatory BP variability in hypertensives.^21^ In the CARDIA study, the lower peak forced vital capacity in young adulthood was an independent risk factor for higher visit‐to‐visit BP variability during middle adulthood.^22^

The carotid bodies, the principal peripheral chemoreceptors, are suggested to be a site of SARS‐CoV‐2 invasion because they are the site of the local expression of the receptor of angiotensin‐converting enzyme (ACE) 2.^23^ Increased peripheral arterial chemosensitivity and reflex SNS overactivation might have been associated with the increased day‐to‐day BP variability in the critically ill COVID‐19 patients reported by Li and coworkers.^1^

Although it is not completely clear whether increased BP variability is a cause or simply an index of decreased arterial compliance,^24^ one major determinant of BP variability depends on the sensitivity of baroreceptor function. Namely, vascular structural changes may reduce baroreceptor sensitivity (BRS) in hypertension.^25^ Decreased large arterial compliance, which contributes to the depressed BRS in young hypertensives,^25^ might enhance the BP fluctuations associated with minor changes in the cardiac stroke volume due to ANS instability.

In the data from the United Kingdom Biobank, the patients who died due to COVID‐19 had significantly higher arterial stiffness as well as significantly lower left ventricular stroke volume compared with those who survived.^26^ SNS overactivity, dysregulation of the renin‐angiotensin system, and altered ACE‐expression are suggested to lead to the adverse structural and functional remodeling of the arteries, that is, linked to the increased large arterial stiffness in patients with COVID‐19.^27^ Moreover, higher SNS activity is associated with vasoconstriction of the coronary microvasculature, and with change in the oxygen demand of cardiomyocytes.^28^ Thus, in the critically ill COVID‐19 patients, increased large arterial stiffness, depressed BRS, and impaired cardiac function are suggested to be pivotal determinants for the exaggerated day‐to‐day BP variability (Figure 1).

**FIGURE 1:**
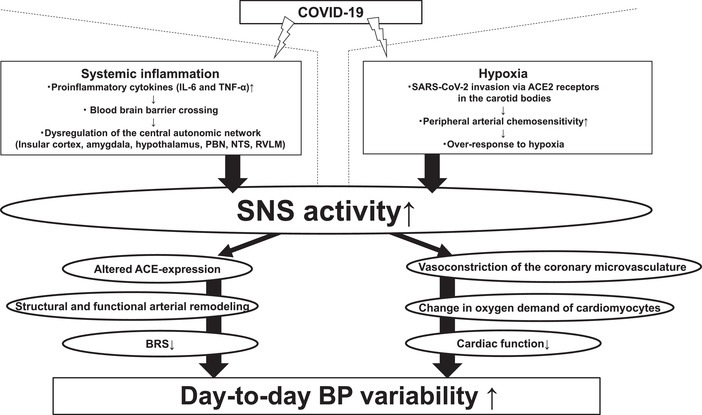
A possible pathway for the relationship between COVID‐19 infection and increased day‐to‐day BP variability. *Abbreviations*: COVID‐19, coronavirus disease 2019; PBN, parabrachial nucleus; NTS, nucleus tractus solitarius; RVLM, rostral ventrolateral medulla; BRS, baroreceptor sensitivity; SARS‐CoV‐2, severe acute respiratory syndrome coronavirus 2; ACE, angiotensin‐converting enzyme; SNS, sympathetic nervous system; BRS, baroreceptor sensitivity; BP, blood pressure

Until now, there have been few reports assessing the relationship between day‐to‐day BP variability and unfavorable outcome in COVID‐19. In addition to strict BP control,^29^, ^30^ it might be important to minimize day‐to‐day BP variability in order to prevent the deterioration of COVID‐19 patients into critical illness. The data presented in the study by Li and coworkers,^1^ thus, make an important contribution, provided that they are considered within the context of the precise pathophysiology underlying the relationship between COVID‐19 infection and day‐to‐day BP variability.

## CONFLICT OF INTEREST

The authors declare no conflict of interest.
